# Associations of Sociodemographic Factors and Health Behaviors with the Emotional Well-Being of Adolescents during the COVID-19 Pandemic in Brazil

**DOI:** 10.3390/ijerph18116160

**Published:** 2021-06-07

**Authors:** Célia Landmann Szwarcwald, Deborah Carvalho Malta, Marilisa Berti de Azevedo Barros, Paulo Roberto Borges de Souza Júnior, Dália Romero, Wanessa da Silva de Almeida, Giseli Nogueira Damacena, André Oliveira Werneck, Danilo Rodrigues Pereira da Silva, Margareth Guimarães Lima, Crizian Saar Gomes, Luiz Otávio Azevedo, Arthur Pate de Souza Ferreira, Renata Gracie, Maria de Fátima de Pina

**Affiliations:** 1 Fundação Oswaldo Cruz, Instituto de Comunicação e Informação Científica e Tecnológica em Saúde, Av. Brasil, 4635-Pavilhão Haity Moussatché-Manguinhos, Rio de Janeiro/RJ-CEP 21045-360, Brazil; paulo.borges@icict.fiocruz.br (P.R.B.d.S.J.); dalia.fiocruz@gmail.com (D.R.); wanessa.almeida@gmail.com (W.d.S.d.A.); damacenagn@gmail.com (G.N.D.); luiz.azevedo@icict.fiocruz.br (L.O.A.); arthurpaterj@gmail.com (A.P.d.S.F.); renata.gracie@icict.fiocruz.br (R.G.); fatima.pina@icict.fiocruz.br (M.d.F.d.P.); 2 Departamento de Enfermagem Materno-Infantil e Saúde Pública, Escola de Enfermagem, Universidade Federal de Minas Gerais, Av. Alfredo Balena, 190 Santa Efigênia, Belo Horizonte/MG-CEP 30130-100, Brazil; dcmalta@uol.com.br (D.C.M.); criziansaar@gmail.com (C.S.G.); 3 Departamento de Saúde Coletiva, Faculdade de Ciências Médicas, Universidade Estadual de Campinas (UNICAMP), Rua Tessália Vieira de Camargo, 126–Cidade Universitária Zeferino Vaz, Campinas/SP-CEP 13083-887, Brazil; marilisa@unicamp.br (M.B.d.A.B.); mglima@unicamp.br (M.G.L.); 4 Departamento de Nutrição, Escola de Saúde Pública, Universidade de São Paulo (USP), Av. Dr. Arnaldo, 715-Cerqueira César, São Paulo/SP CEP 01246-904, Brazil; andreowerneck@gmail.com; 5 Departamento de Educação Física, Universidade Federal de Sergipe, Av. Marechal Rondon, s/n-Jardim Rosa Elze, São Cristóvão/SE CEP 49100-000, Brazil; danilorpsilva@gmail.com

**Keywords:** adolescents, social isolation, emotional well-being, sociodemographic factors, unhealthy behaviors, COVID-19 pandemic, Brazil

## Abstract

This cross-sectional study utilizes data from a nationwide web-based survey aimed to identify the factors affecting the emotional well-being of Brazilian adolescents aged 12–17 during the period of school closures and confinement. Data collection took place from 27 June to 17 September 2020. We used the “virtual snowball” sampling method, and students from private and public schools were included. A total of 9470 adolescents were analyzed. A hierarchical logistic regression model was used to find the factors associated with reporting at least two of three self-reported problems—sadness, irritability, and sleep problems. The main proximal factor was loneliness (AdjOR = 8.12 *p* < 0.001). Problems related to school closures also played an important role. Regular intake of fruits and vegetables, as well as physical activity, demonstrated a positive influence on emotional well-being, while excessive screen time (AdjOR = 2.05, *p* < 0.001) and alcohol consumption negatively affected outcomes (AdjOR = 1.73, *p* < 0.001). As for distal variables, less affluent adolescents were the most affected, and males reported fewer emotional problems than females. Uncertainty regarding the disease in a context of socioeconomic vulnerability, together with rises in unhealthy behaviors and isolation from their immediate social circles, have negatively affected adolescents’ emotional status throughout the COVID-19 pandemic.

## 1. Introduction

The global COVID-19 pandemic has forced the implementation of drastic measures to control the massive spread of the virus. As well as timely diagnosis, case isolation, and large-scale case screening, public health measures to contain the disease spreading include lockdown and other social restraint measures [1]. 

The arrival of the disease caused by SARS-COV-2 in Brazil in February 2020, led to a series of initiatives and recommendations for the population’s protection. Since the first deaths due to the disease in Brazil, public health measures such as home confinement and restraints on human contact have been adopted in the larger Brazilian cities, and lockdowns of smaller cities have also been implemented to prevent the entry of cases from metropoles. Schools and non-essential businesses have been closed, workers have been instructed to carry out activities at home, travel has been restricted, and some cities have closed borders to reduce circulation between cities [2].

Nevertheless, despite the effectiveness of these social restraining measures to contain transmission and prevent the overload of health systems [3,4], the guidance pertaining to home confinement for people who do not exercise essential occupations has had relevant implications in a socioeconomic context, with a significant reduction in family incomes, especially among socially disadvantaged people, aggravating social and health inequalities [5].

As well as the socioeconomic impact, facing social isolation is a challenging experience. Separation from loved ones, loss of freedom, uncertainty about the disease, and changes in routine activities have all led to distress, anxiety, and depression, and have generated psychological problems [6,7,8,9,10] and the adoption of unhealthy behaviors [11,12,13] in several countries. 

Results of the first stage of the ConVid-Behavior Survey carried out in the Brazilian adult population have shown the negative impact of pandemic-related social restraint measures on the overall health of citizens who have been affected by psychological health problems [14,15]. However, there were differences in the results by age group, with older adults (60 years or over) reporting less anxiety, sadness, and loneliness in comparison to the youngest group (18–29 years old). Moreover, sedentary lifestyles and the consumption of sweets and chocolates were more prevalent among young people [16]; similar findings have been found in other studies [17,18].

Evidence in the adult Brazilian population suggests that COVID-19’s effects on adolescents’ well-being are likely to be intense. As with young adults, children and adolescents are confined to their homes without outdoor activities or face-to-face classes. A study in Germany showed that children and adolescents’ experiences could not be separated from those of their families. As well as experiencing more mental health problems and higher anxiety levels than before the pandemic, teenagers with a low socioeconomic status, a migration background, and a limited living space were significantly more affected [19]. 

Findings from systematic reviews showed that the COVID-19 pandemic has had an important impact on adolescents’ mental health [20,21]. Anxiety, depression, loneliness, and poor sleep quality are the most common psychological problems [22,23,24,25]. Several modifiable risk and protective factors have been identified in adolescents, with better nutritional status and regular practice of physical activity associated with lower levels of depressive and anxiety symptoms, as well as better sleep quality [26,27], whereas alcohol consumption was associated with higher levels of mental health problems [28]. Moreover, evidence of socioeconomic inequalities has been found for both mental health and healthy lifestyles, and it invariably shows unfavorable data regarding adolescents of disadvantaged families [19,29].

During the pandemic, data on youth mental health have started to accumulate [30]. However, studies in Brazil among adolescents are still sparse. Studies with college students in two Brazilian cities showed a high prevalence of symptoms of stress, depression, anxiety, and mood swings. In addition, engagement in physical activity was associated with better psychological outcomes [31,32]. 

To the best of our knowledge, ours is the first cross-country survey among Brazilian adolescents during the COVID-19 pandemic. The aim of the current study was to investigate the associations between adolescents’ emotional well-being and problems brought about by the social restraint measures, and whether family affluence and healthy lifestyles contribute to these associations. It is hypothesized that higher levels of lifestyle behavior are associated with better emotional well-being, and that lower socioeconomic status (SES) is associated with lower levels of healthy lifestyles and poorer emotional health [33]. Our results may indicate which lifestyle behaviors are associated with adolescent health, and support interventions for mitigating pandemic-related adverse effects, not only in the current outbreak but in possible future events. 

## 2. Methods

The “ConVid—Adolescents” is a cross-sectional study on adolescent behaviors during the COVID-19 pandemic in Brazil. This is the second part of the “ConVid—Behavior Survey” (https://convid.fiocruz.br/, accessed on 21 April 2021) carried out among Brazilian adults. The nationwide web survey was conducted by the Oswaldo Cruz Foundation, in partnership with the Federal University of Minas Gerais and the State University of Campinas. The project was approved by the National Research Ethics Commission (Process number 3.980.277).

For collection and management of the data, the RedCap (Research Electronic Data Capture) application was used. Data collection took place from 27 June to 17 September 2020. The questionnaire was filled out by the respondent using a cell phone or laptop/computer with internet access after consent was given by the adolescent’s parent or guardian and the adolescents themselves consented. Copies of the documents for free and informed consent are available on the research site (https://convid.fiocruz.br/, accessed on 21 April 2021). All the responses were anonymous and without any other identification of the participants. The information was directly stored on the server of the Institute of Communication and Scientific and Technological Information in Health of the Oswaldo Cruz Foundation (ICICT/FIOCRUZ). Inclusion criteria were the following: 12–17 years old and being a Brazilian citizen residing in the country during the pandemic. 

The questionnaire had 54 multiple-choice items. The questions addressed demographic characteristics, possession of a cell phone, computer, or notebook, and access to the internet at home, as well as indicators of family socioeconomic status. The items related to the pandemic addressed the difficulties of following non-face-to-face classes, whether the adolescent or a relative had COVID-19, adherence to social restraint measures, hygiene behaviors, and positive and negative experiences during the pandemic. To test the validity and reliability of the questionnaire, a pre-test was carried out with 20 adolescents living in different states and from different types of schools (public and private).

Besides questions formulated specifically for the questionnaire (DOI: 10.7303/syn23583473.1), questions validated in previous health surveys in Brazil were also used. Items regarding lifestyles were based on questions from the National School Health Survey (PeNSE, 2015) [34]. The following health behaviors were addressed: physical activity, sedentarism, the consumption of healthy and unhealthy foods, smoking, and the use of alcohol. 

The questions on emotional problems were adapted from the World Health Survey [35] and, to evaluate sleep quality, a single question regarding sleep self-assessment was used, which was adapted from the set of questions on sleep problems from the Population-Based Health Survey in Campinas, SP, 2014–2015 [36]. 

As part of a set of items to evaluate adolescents’ emotional well-being during the COVID-19 pandemic, the following three questions were asked: “In the period of confinement how often did you feel sad?”; “In the period of confinement how often did you feel irritated?”, the responses to which were on a 5-point Likert scale (never, rarely, sometimes, often, and always); and “Did the pandemic affect the quality of your sleep?”, with the following response options: no sleep problems, got better, as usual, started to have sleep problems, and got worse. The dichotomous outcome, “Presence of emotional problems”, was defined by the presence/absence of at least two of the following problems—frequent sadness, frequent irritability, and worsening of sleep quality during COVID-19.

### 2.1. Sampling

We used the “virtual snowball” sampling method, which began by sending invitations through the electronic questionnaire link by the virtual social network WhatsApp or by e-mail. First, researchers selected teenager’s parents from different Brazilian states to start the process. In addition, public and private schools were invited to participate through a Fiocruz institutional e-mail. The schools that decided to participate in the survey sent the electronic questionnaires to their students. After completing the questionnaire, the adolescents invited other adolescents through social networks, thus making up the recruitment chain. At the end of the data collection (27 June to 17 September 2020), the sample reached 9470 adolescents aged 12 to 17 years from all Brazilian states.

### 2.2. Data Analysis

The sample was weighted using data from the National School Health Survey (PeNSE, 2015) [34] from the Brazilian Institute of Geography and Statistics (IBGE) to obtain the same sample distribution by macro-region, sex, age group (12–15; 16–17), skin color, and type of school (public/private).

To analyze the factors associated with the outcome, we used a hierarchical model of determination [37]. This type of analysis considers the association of each independent variable with the response variable, controlling for possible confounder effects between proximal and distal variables. Under the hypotheses that higher levels of healthy behavior are associated with better emotional well-being, and that low SES is associated with lower levels of healthy behaviors and poorer emotional health [33], our conceptual model was developed to integrate social and behavioral indicators to explain their relationship to adolescent emotional well-being during the COVID-19 pandemic.

The model includes factors that have traditionally been identified as relevant to the outcome, including sociodemographic variables [38] and lifestyle indicators [39], as well as other problems closely related to the COVID-19 pandemic. The independent variables were organized into three hierarchical blocks (one distal, one intermediary, and one proximal): sociodemographic characteristics; lifestyle indicators; and problems that occurred during the pandemic. The hierarchy of variables was maintained throughout the statistical analysis.

The proximal variables (block 3), which may affect the outcome directly were: adolescent had COVID-19; a family member or close friend had COVID-19; adherence to social restrain measures (stayed at home all the time or most of the time); frequent loneliness (often felt lonely during home confinement); missed school (missed going to school during home confinement); concentration difficulties in non-face-to-face classes (lack of concentration was one of the difficulties to follow non-face-to-face classes). 

Hierarchically, we considered behavior indicators during the pandemic which may have affected an adolescent’s emotional status through intermediate mechanisms [39] (block 2). The following behavior indicators during the COVID-19 pandemic were considered in the analysis: alcohol consumption; intake of at least one unhealthy food (pizza, sausages, frozen hamburger, sweets, and chocolates, soft drinks, snacks) five or more days per week; vegetable and fruit intake five or more days per week; one hour of physical activity at least twice a week; and excessive entertainment screen time (≥6 h/day) (including computer, tablet, cell phone, and television screens). 

Lastly, sociodemographic characteristics were the distal determinants, which may interfere in both the adoption of healthy behaviors and in emotional well-being [40]. In block 1, the following sociodemographic characteristics were considered: sex, age groups (12–15; 16–17 years old), skin color (white; non-white), mother’s educational level (no college education, complete college education), family financial difficulties, and food insecurity (“Was there ever a concern that food would run out before your parents had money to buy more food?”).

Initially, the blocks were analyzed separately. For each block of variables, a descriptive analysis of each variable was carried out and the outcome prevalence was estimated for each variable category. To assess the possible factors associated with the outcome, we used logistic regression models at a 5% significance level to test the associations with the covariates of each hierarchical block. The odds ratio (OR) was used as the measure of association. 

For the multivariate analysis, the variable blocks were inserted sequentially and hierarchically, starting with the sociodemographic block, the behavior indicators and, lastly, the pandemic-related variables. We used the adjusted OR to test, at the 5% significance level, the associations with the block covariates, controlling for the variables of the previous block. The variables with *p*-values < 0.05 in each block analysis were included in the multivariate model. In the final multivariate analysis, the variables with *p*-values < 0.05 remained. 

## 3. Results

A total of 9470 adolescents were analyzed, of whom 49.8% were males and 50.2% were females; 67.7% of them were 12–15 years old (Table 1). Regarding the self-reported problems, 23.2% reported being frequently sad, and 9.2% had always felt sad. As for irritability, 29.4% showed frequent irritability and 19.4% had always felt irritated. Regarding sleep quality, 23.9% started to have sleep problems and 12.1% reported a worsening of sleep problems. The prevalence of at least two of those problems was 37.6%, with 15.5% presenting all three problems. 

In Table 2, we analyze the associations between the sociodemographic factors and the outcome. The prevalence of two or more self-reported emotional problems was significantly higher among females (50.1%) than males (24.8%). Regarding age groups, the outcome was significantly and directly correlated with age: the lower the age, the lower the outcome prevalence, ranging from 34.3% among the youngest (12–15 years old) to 44.5% among the older adolescents (16–17 years old). More than one-fifth of adolescents reported family financial difficulties and food insecurity. Financial difficulties showed a significant association with the outcome, with 1.40 greater odds of being emotionally affected than adolescents from more affluent families. A lower but significant odds ratio (1.12) was also found for food insecurity. Small differences were found for skin color. As for the mother’s educational level, no significant association was found.

Considering unhealthy habits during the pandemic (Table 3), the prevalence of alcohol consumption was 12.8% and the prevalence of unhealthy food intake 5 days or more/week was 30.5%. For 46.4% of adolescents, entertainment screen time was greater or equal to 6 h/day. As for healthy behaviors, the prevalence of vegetable and fruit intake 5 days/week was 19.6%, and 41.8% engaged in one or more hours of physical activity at least twice a week. 

Associations of the outcome with lifestyle indicators were also examined in Table 3. Physical activity for 60 min or over at least twice a week was inversely correlated with the outcome (OR = 0.82, *p* < 0.001), as well as regular intake of fruits and vegetables (OR = 0.72; *p* < 0.001). Direct and significant associations with unhealthy behaviors were found, with higher odds among teenagers with ≥6h/d screen time (OR = 2.57, *p* < 0.001), those with alcohol consumption (OR = 2.10, *p* < 0.001), and among adolescents with unhealthy food intake at least 5 days a week (OR = 1.50, *p* < 0.001). After controlling for the first block of sociodemographic variables, significantly adjusted ORs were found for all unhealthy behaviors, while regular intake of vegetables and fruits and engagement in physical activity were protective factors.

As for the COVID-19-related variables, 71.5% adhered to social protection measures, 73.0% missed school, 59.0% reported difficulties in concentrating in non-face-to-face classes, 32.8% frequently felt lonely, 4.0% had COVID-19, and, for 43.2% of the participants, a family member or close friend had COVID-19 (Table 4).

Considering the set of proximal variables, loneliness was identified as the factor most associated with the outcome, with approximately 10 times greater odds of reporting emotional problems among adolescents with frequent lonely feelings. Both school closure-related indicators were associated with the outcome, with significant odds-ratios for concentration difficulties in non-face-to-face classes (OR = 2.35, *p* < 0.001) and in terms of missing school (OR = 1.93, *p* < 0.001). Having had COVID-19 was significantly associated with the response variable (OR = 1.83, *p* < 0.001), as was a family member or a close friend having had COVID-19 (1.77, *p* < 0.001). Adherence to social restriction measures also showed a significant association with the outcome (OR = 1.62, *p* < 0.001). The effects of all proximal variables remained significant, even after controlling for sociodemographic variables and lifestyle indicators (Table 4). 

The results of the final multivariate analysis are presented in Table 5. After controlling for all 5% level significant variables for the three hierarchical blocks, loneliness remained the most associated factor with the outcome. Significant effects of the three unhealthy habits were also found, with 6 h or more screen time and alcohol consumption showing the highest adjusted odds-ratios. Both healthy behaviors, regular intake of fruits and vegetables, and at least 1 h of physical activity 2 or more days per week showed significant protective effects. Among the sociodemographic factors, differences by sex and family financing difficulties remained significant.

## 4. Discussion

In this study, a hierarchical logistic regression model was used to identify the factors associated with the self-reporting of at least two from the following problems—frequent sadness, frequent irritability, and worsening of sleep quality during the COVID-19 pandemic. The main proximal factor was loneliness, but problems related to school closure also played an important role. As for the intermediation of behavioral indicators, the intake of healthy food and practice of physical activity during the pandemic showed a positive influence on emotional well-being, while excessive screen time negatively affected the outcome. In turn, associations with the distal variables corroborate previous results [5], with less affluent adolescents being the most affected and females reporting problems more often than males. 

During the period of social restraint measures, low-income families had more difficulties in facing the pandemic [40]. In Brazil, there was a greater reduction in family income among the poorest sections of the population, while informal workers were left without work, raising concerns about family maintenance and food insecurity [41,42,43]. The findings of this study showed adolescents from disadvantaged families are more likely to have emotional problems, probably due to uncertainty and anxiety among adolescents regarding their families’ financial difficulties [44]. The negative emotional consequences indicate the need to intensify mental health protection actions, giving priority to the most vulnerable families [43].

In addition, the closure of private and public schools, parks, and recreation centers resulted in changes in the overall mood of Brazilian adolescents, and sadness, irritability, and sleep problems have become quite usual. Impaired emotional status during the COVID19 pandemic has been previously shown by several studies and has been attributed to a disruption in education, physical activities, and socialization [45,46]. 

A systematic review of recent studies showed that anxiety, depression, irritability, mood swings, and sleep problems were often found during lockdown or social isolation periods [25]. In Brazil, the most prevalent problem was irritability, with almost half of the participants reporting frequent irritability. It is interesting to note that irritability is defined as an emotional state that tends to lead to anger in response to frustration [47]. In the context of the COVID-19 pandemic, irritability is likely to reflect the frustration of not being able to enjoy routine and amusement activities. Frequent sadness was reported by one-third of the participants, which was probably motivated by loneliness and a lack of friends, teachers, and relatives in most cases. As for sleep quality, almost a quarter of adolescents started having sleep problems, and more than 10% reported a worsening of these problems, indicating that adolescents have been especially vulnerable to disturbed sleep during this period of change and uncertainty.

In this study, emotional issues were significantly more common among females. As has been documented before, anxiety and depressive symptoms are generally more common among women [48], while men have a greater misunderstanding of depression [49]. The findings also revealed frequent sedentary behaviors, unhealthy food intake, alcohol consumption, and reduction of physical activity during the COVID-19. All these unhealthy habits showed negative associations with worsening of emotional status, corroborating results of other publications [26,27,28,50]. On the other hand, the regular intake of fruits and vegetables as well as the practice of physical activity during the pandemic showed a positive influence on emotional well-being in line with previous evidence [51]. Efforts are being made to encourage the practice of physical activities in Brazil [52]. Home confinement, however, caused a substantial decrease in physical activity. Prolonged bouts of sedentary behavior result in various health risks, including increased levels of stress, depression, and anxiety [53]. This is in contrast to engaging in regular physical activity, which provides a feeling of well-being and relieves tension [54]. Additionally, in this study, excessive screen time was highly associated with the presence of emotional problems among Brazilian adolescents. Although rises in screen time may be inevitable for education and socialization during home confinement, excessive screen time increases sedentary time and is associated with health risks [55]. The negative consequences on psychological problems are also well-documented [56].

Unfortunately, the emergence of the second and third waves of new SAR-COV-2 variants in Brazil has once again forced municipal and state governments to impose strict social restraint measures. Parents’ efforts to reduce their children’s unhealthy eating habits and sedentary behaviors, helping them to adapt to changes in school activities, and seeking indoor activities that may provide fun stress relief will all be useful to mitigate adverse health effects in the coming days.

One limitation of our study was the assessment of adolescents’ emotional states by relying on single item self-evaluations, which generally produce less reliable results than multi-item scales [57]. Other limitations refer to the virtual snowball sampling, which is a non-probabilistic sampling technique. Adolescents who do not have access to the internet by either cell phone or computer were not able to participate in the survey. The dependence of observations resulting from our chain recruitment may also have caused bias in the estimates and statistical inference results [58]. In addition, bias in causal associations inherent to cross-sectional studies cannot be disregarded [59]. Moreover, psychological well-being or diagnosis of any mental disorder before the pandemic were not considered, which could confound data interpretation in terms of temporality.

## 5. Conclusions 

The uncertainty about the COVID-19 pandemic’s progression in a context of socioeconomic vulnerability, together with rises in unhealthy behaviors and isolation from immediate social circles, are negatively affecting Brazilian adolescents’ emotional well-being. Efforts focused on reducing unhealthy and sedentary behaviors, such as excessive screen time and the promotion of indoor activities that may provide fun, socialization, and physical activity will be useful for improving adolescents’ emotional health.

## Figures and Tables

**Table 1:** Prevalence (%) of self-reported sadness, irritability, and sleep problems among adolescents during the COVID-19 pandemic. Brazil, 2020.

Problems		Sample Size	Prevalence (%)
Sadness	Never/seldom	3142	33.2
	Sometimes	3220	34.2
	Often	2179	23.2
	Always	869	9.2
Irritability	Never/seldom	1893	20.1
	Sometimes	2929	31.1
	Often	2763	29.4
	Always	1820	19.4
Sleep problems	Never	4996	53.2
	As usual	1016	10.8
	Started to have	2245	23.9
	Got worse	1140	12.1
Number of problems	0	3352	35.8
	1	2491	26.6
	2	2066	22.1
	3	1453	15.5
At least two problems *	Less than 2	5844	62.4
	2 or more	3518	37.6

* At least two problems from frequent sadness, frequent irritability, and sleep problems.

**Table 2:** Prevalence of sociodemographic characteristics and associations with the outcome * during the COVID-19 pandemic. Brazil, 2020.

Variable	Category	%	Outcome Prevalence (%)	OR ^1^	*p*-Value ^2^
Sex	F	50.2	50.1	1.00	-
	M	49.8	24.8	0.33	<0.001
Age group	12–15	67.7	34.3	1.00	-
	16–17	32.3	44.5	1.48	<0.001
Skin Color	White	40.1	36.0	1.00	<0.001
	Non White	59.9	40.1	1.19	-
Mother’s educational level	Complete high school	69.3	37.3	1.00	-
	Complete college education	30.7	38.2	1.04	0.449 (NS)
Family financial difficulties	Yes	23.1	43.8	1.40	<0.001
	No	76.9	35.8	1.00	-
Food insecurity	Yes	26.1	39.6	1.12	0.018
	No	73.9	36.9	1.00	-

* At least two problems from frequent sadness, frequent irritability, and sleep problems. ^1^ Odds ratio (OR). ^2^ Level of significance of the OR test.

**Table 3:** Prevalence (%) of lifestyle indicators and associations with the outcomes * during the COVID-19 pandemic. Brazil, 2020.

Indicator	Category	Indicator Prevalence (%)	Outcome Prevalence (%)	Crude OR	*p*-Value	Adjusted OR ^1^	*p*-Value ^2^
Alcohol consumption	Yes	12.8	53.5	2.10	<0.001	1.73	<0.001
	No	87.2	35.4	1.00	-	1.00	-
Screen time (≥6 h/day)	Yes	46.4	49.3	2.57	<0.001	2.51	<0.001
	No	53.6	27.4	1.00	-	1.00	-
At least 1 h physical activity twice a week	No	58.2	39.5	1.00	-	1.00	-
	Yes	41.8	35.0	0.82	<0.001	0.88	0.004
Unhealthy food consumption ≥5 days a week	Yes	30.5	39.1	1.50	<0.001	1.24	<0.001
	No	69.5	31.7	1.00	-	1.00	-
Vegetable and fruit intake ≥5 days a week	Yes	19.6	31.7	0.72	<0.001	0.80	<0.001
	No	80.4	39.1	1.00	-	1.00	-

* At least two problems from frequent sadness, frequent irritability, and sleep problems. ^1^ Adjusted odds ratio (OR)—controlling for sex, age group, skin color, family financial difficulties, and food insecurity. ^2^ Level of significance of the adjusted OR test.

**Table 4:** Prevalence (%) of pandemic-related indicators and associations with the outcome * during the COVID-19 pandemic. Brazil, 2020.

Indicator	Category	%	Prevalence	Crude OR	*p*-Value	Adjusted OR ^1^	*p*-Value ^2^
Adherence to social restriction	Yes ^3^	71.5	40.7	1.62	<0.001	1.21	0.002
	No	28.5	29.8	1.00	-	1.00	-
Missed school	Yes ^4^	73.0	41.5	1.93	<0.001	1.46	<0.001
	No	27.0	26.9	1.00	-	1.00	-
Concentration difficulties in non-face-to-face classes	Yes ^5^	59.0	45.5	2.35	<0.001	1.61	<0.001
	No	41.0	26.2	1.00	-	1.00	-
Frequent loneliness	Yes ^6^	32.8	71.9	9.74	<0.001	8.12	<0.001
	No	67.2	20.8	1.00	-	1.00	-
Adolescent had COVID-19	Yes	4.0	51.7	1.83	<0.001	1.58	0.001
	No	96.0	37.0	1.00	-	1.00	-
Family member or close friend had COVID-19	Yes	43.2	45.2	1.77	<0.001	1.46	<0.001
	No	56.9	31.8	1.00	-	1.00	-

* At least two problems from frequent sadness, frequent irritability, and sleep problems. ^1^ Adjusted odds ratio (OR)—controlling for sex, age group, skin color, family financial difficulties, food insecurity, and health behaviors (alcohol consumption, screen time (≥6 h/day), at least 1 h physical activity twice a week, unhealthy food consumption ≥5 days a week, and vegetable and fruit intake ≥5 days a week). ^2^ Level of significance of the adjusted OR test. ^3^ Stayed at home all the time or most of the time. ^4^ Missed going to school during home confinement. ^5^ Lack of concentration was one of the difficulties in following non-face-to-face classes. ^6^ Often felt lonely during the COVID-19 pandemic.

**Table 5:** Most associated factor with the outcome * during the COVID-19 pandemic. Brazil, 2020.

Indicator	Category	Adjusted OR ^1^	*p*-Value ^2^
Sex	F	1.00	-
	M	0.41	<0.001
Family financial difficulties	Yes	1.36	<0.001
	No	1.00	-
Alcohol consumption	Yes	1.73	<0.001
	No	1.00	-
Screen time (6 h or over a day)	Yes	2.05	<0.001
	No	1.00	-
At least 1 h physical activity twice a week	No	1.00	-
	Yes	0.87	0.013
Unhealthy food consumption ≥5 days a week	Yes	1.20	0.002
	No	1.00	-
Vegetable and fruit consumption ≥5 days a week	Yes	0.84	0.011
	No	1.00	-
Adherence to social restriction	Yes ^3^	1.21	0.002
	No	1.00	-
Missed school	Yes ^4^	1.46	<0.001
	No	1.00	-
Concentration difficulties in non-face-to-face classes	Yes ^5^	1.61	<0.001
	No	1.00	-
Frequent loneliness	Yes ^6^	8.12	<0.001
	No	1.00	-
Adolescent had COVID-19	Yes	1.58	0.001
	No	1.00	-
Family member or close friend had COVID-19	Yes	1.46	<0.001
	No	1.00	-

* At least two problems from frequent sadness, frequent irritability, and sleep problems. ^1^ Adjusted odds ratio (OR)—controlling for all variables in the model. ^2^ Level of significance of the adjusted OR test. ^3^ Stayed at home all the time or most of the time. ^4^ Missed going to school during home confinement. ^5^ Lack of concentration was one of the difficulties in following non-face-to-face classes. ^6^ Often felt lonely during the COVID-19 pandemic.

## Data Availability

Information about the web survey can be found at https://convid.fiocruz.br/, (accessed on 21 April 2021) and the database supporting the reported results is available upon request.
